# Faecal immunochemical test for suspected colorectal cancer symptoms: patient survey of usability and acceptability

**DOI:** 10.3399/BJGPO.2021.0102

**Published:** 2022-01-12

**Authors:** Theo Georgiou Delisle, Nigel D'Souza, Bethan Davies, Sally Benton, Michelle Chen, Helen Ward, Muti Abulafi

**Affiliations:** 1 Croydon University Hospital, London, UK; 2 Imperial College London, London, UK; 3 Basingstoke and North Hampshire Hospital, Basingstoke, UK; 4 Royal Surrey County Hospital, Guildford, UK; 5 RM Partners, The West London Cancer Alliance, London, UK

**Keywords:** patients, colorectal neoplasms, colonoscopy, perception, faeces, general practice

## Abstract

**Background:**

Recent evidence suggests that the faecal immunochemical test (FIT) can rule out colorectal cancer (CRC) in symptomatic patients. To date, there is no research on usability and perception of FIT for these patients.

**Aim:**

To measure variation in attitudes and perception of FIT in patients with suspected CRC symptoms.

**Design & setting:**

A cross-sectional survey of a subset of participants of the NICE FIT study.

**Method:**

A questionnaire was co-developed with patients covering four themes on a Likert scale: FIT feasibility, faecal aversion, patient knowledge, and future intentions. Questionnaire and FIT kits were sent to patients with suspected CRC symptoms participating in the NICE FIT study. Logistic regression explored differences in patients’ test perception by ethnic group, language, age, location, deprivation, FIT use, and previous experience.

**Results:**

A total of 1151 questionnaires were analysed; 90.2% (95% confidence interval [CI] = 88.3% to 91.8%) of patients found faecal collection straightforward, 76.3% (95% CI = 73.7% to 78.6%) disagreed FIT was unhygienic, and 78.1% (95% CI = 75.6% to 80.4%) preferred FIT to colonoscopy. Preference for FIT over colonoscopy was weaker in patients aged 40–64 years than those >65 years (odds ratio [OR] 0.60; 95% CI = 0.43 to 0.84). Intention to use FIT again was stronger in patients who successfully used FIT than those unsuccessful (OR 11.08; 95% CI = 2.74 to 44.75), and white compared with non-white patients assessed (OR 3.20; 95% CI = 1.32 to 7.75).

**Conclusion:**

While most patients found FIT practical and hygienic, perception differences were found. Strategies to engage patients with more negative FIT perception should underpin symptomatic FIT pathways.

## How this fits in

This article is the first study to report on attitudes to FIT from patients who have personally experienced potential CRC symptoms and been offered FIT. Most symptomatic patients who responded found using FIT was acceptable and did not generate negative feelings of faecal aversion. However, perception differences were found between patient groups that should inform future FIT pathways in primary and secondary care to improve patient experience.

## Introduction

FIT is a non-invasive, quantitative immunoassay that detects the globin moiety of haemoglobin in faeces (f-Hb). FIT is used in >25 bowel screening programmes worldwide.^1^ In 2017, NICE recommended FIT use in primary care to triage patients with low-risk symptoms for CRC before referral;^2^ however, this was not extended to include high-risk symptoms for CRC referred under the 2-week wait (2WW) pathway.^3^ There is mounting evidence of the high diagnostic accuracy of FIT in these patients^4,5^ and it is likely FIT will be introduced nationally to triage patients for referral. The need to streamline endoscopy services during the COVID-19 pandemic has further shifted emphasis towards using FIT to guide 2WW referrals.^6^

Research on patient acceptability and perception of faecal tests has focused on asymptomatic individuals in screening programmes.^7–10^ The main focus of these studies was to understand reasons for poor test uptake, which could affect the screening programme efficacy. However, although FIT uptake was high when used to prioritise investigation as part of a service evaluation of a patient referral pathway investigating worrying symptoms of suspected CRC,^11^ uptake was lower in research studies of patients with similar symptoms where FIT did not guide patient care.^12,13^ This indicates the need for better understanding of the variation of patient perception and attitudes to FIT when used in urgent referral pathways, to inform design of future pathways in primary and secondary care, and improve patient experience. The study focused on patients referred under the 2WW pathway with suspected CRC symptoms recruited into the NICE FIT study, a multicentre, double-blinded diagnostic accuracy study in 50 English NHS hospitals, which determined the sensitivity and specificity of FIT for CRC when compared with colonoscopy.^12^ The aim was to determine variation in attitudes, perception, and usability of FIT in these symptomatic patients.

## Method

### Questionnaire co-production with public and expert input

A literature review was carried out to develop a patient FIT questionnaire with input from a patient panel (Cancer Research UK) and the NICE FIT Steering Committee. Questionnaire items were drawn from previously published patient questionnaires (16 in total) relating to faecal tests; however, there was no single published validated questionnaire assessing all items that were used. Questionnaire format was a series of statements that participants could respond to using a 5-point Likert scale (strongly agree, agree, neither, disagree, strongly disagree). Twenty-one statements were generated covering four themes: feasibility of FIT, patient feelings of faecal aversion towards FIT, knowledge in relation to bowel cancer, and future test intentions. The questionnaire also collected demographic information and previous test experience. Questionnaire statements used in the study are shown in Table 1.

**Table 1.** Questionnaire statement’s relationship to patient themes. Patients were asked to respond to each questionnaire statement on a 5-point Likert scale (strongly agree, agree, neither, disagree, strongly disagree).

Questionnaire statement	Theme
I found the instructions easy to understand	Feasibility of FIT
I found the device easy to open and close	
I found it straightforward to collect my stool sample	
I would recommend the test to others	
I would prefer to complete a FIT kit rather than go straight for colonoscopy	
I feel confident about collecting a stool sample	
The FIT test is unpleasant	
It is difficult to find time to do the stool test	
I am happy for the FIT kit to be sent by post rather than through my GP	
Collecting a stool sample to detect bowel cancer is unhygienic	Faecal aversion towards FIT
It is difficult to overcome the disgust related to the stool test	
It is difficult to overcome the embarrassment related to the stool test	
I can name some symptoms of bowel cancer	Knowledge in relation to bowel cancer
I think that if bowel cancer is in your family it increases your risk of getting it too	
If detected early I think there is a good chance bowel cancer can be cured	
I am worried about getting bowel cancer	
I would be prepared to use the FIT kit again in the future	Future test intentions
I understand what the test is being used for	
I think about the future of my health and this influences my behaviour today	
The ability of the test to detect cancer is important for me in making the decision to complete the test	
The ability of the test to detect pre-cancerous lumps is an important factor for me in making the decision to complete the test	

FIT = faecal immunochemical test.

### Data collection

The questionnaire was disseminated to participating NHS hospital trusts across England as a substudy to the NICE FIT study, and 25 sites recruited patients between December 2018 and July 2019. Eligible patients were those referred from primary care with suspected CRC symptoms under the 2WW pathway, triaged for colonoscopy and who were able to complete and return the questionnaire. Questionnaire and instructions were in English. To recruit, patients who agreed to take part in the NICE FIT study were invited to take part in the substudy and were sent the questionnaire alongside a FIT kit, and asked to return it together with completed FIT in a prepaid envelope. All patients who returned the questionnaires were included in the study, irrespective of whether they returned the FIT kit for analysis or not. Patients who were not referred under the 2WW pathway or were not triaged to colonoscopy were not eligible.

Over 3000 questionnaire packs were sent out containing the FIT kit (HM-JACKarc, Kyowa [now Hitachi], Japan), test instructions, and questionnaire. Completed FIT kits and questionnaires were returned by post to the Bowel Cancer Screening Southern Programme Hub. Linked laboratory analysis of kits allowed a comparison of questionnaire responses with correct test use (a returned FIT that could be analysed to produce an f-Hb result). Study consent was through return of the questionnaire, as approved by the Confidentiality Advisory Group (CAG).

### Analysis

Statistical analysis was performed using IBM SPSS Statistics (version 27). The questionnaire Likert scale responses are presented in full in Table 2, but then converted into binary responses for statistical analysis: positive (strongly agree, agree) and non-positive (neutral, disagree, strongly disagree). Covariates were categorised into binary variables (ethnic group: white or non-white; deprivation index: more deprived deciles 1–5 or less deprived deciles 6–10; sex: male or female; preferred language: English or other; location: London or outside of London; test used properly: yes or no; previous stool test experience: yes or no). Age was categorised by groups: 25–39, 40–64, and >65 years, with the older group used as the reference for comparison. Key dependent variable question responses included in this analysis were selected in collaboration with the NICE FIT Steering Committee with expert and public involvement (Cancer Research UK), as representative of questionnaire themes. Proportions are presented with 95% CIs calculated using the Public Health England tool.^14^ Binary logistic regression was used to explore demographic factors influencing patient responses.

**Table 2.** Patient responses to questionnaire statements

Questionnaire statement	Strongly agree, *n*	Agree, *n*	Neutral, *n*	Disagree, *n*	Strongly disagree, *n*	Total, *n*	% positive or negative^a^	95% CI
I found the instructions easy to understand	644	462	23	9	10	1148	96.3	95.1 to 97.3
I found the device easy to open and close	651	441	26	16	5	1139	95.9	94.6 to 96.9
I found it straightforward to collect my stool sample	503	524	64	40	8	1139	90.2	88.3 to 91.8
I would recommend the test to others	706	374	56	7	5	1148	94.1	92.6 to 95.3
I would prefer to complete a FIT kit rather than go straight for colonoscopy	614	280	171	57	23	1145	78.1	75.6 to 80.4
I feel confident about collecting a stool sample	537	516	69	23	3	1148	91.7	90.0 to 93.2
The FIT test is unpleasant	25	146	241	430	301	1143	64.0	61.1 to 66.7
It is difficult to find time to do the stool test	22	70	136	494	420	1142	80.0	77.6 to 82.3
I am happy for the FIT kit to be sent by post rather than through my GP	513	520	84	11	14	1142	90.5	88.6 to 92.0
Collecting a stool sample to detect bowel cancer is unhygienic	19	67	186	479	395	1146	76.3	73.7 to 78.6
It is difficult to overcome the disgust related to the stool test	23	62	179	439	446	1149	77.0	74.5 to 79.4
It is difficult to overcome the embarrassment related to the stool test	22	55	162	464	445	1148	79.2	76.7 to 81.4
I can name some symptoms of bowel cancer	204	593	168	117	34	1116	71.4	68.7 to 74.0
I think that if bowel cancer is in your family it increases your risk of getting it too	276	569	231	43	6	1125	75.1	72.5 to 77.5
If detected early I think there is a good chance bowel cancer can be cured	423	628	76	2	1	1130	93.0	91.4 to 94.4
I am worried about getting bowel cancer	352	529	198	41	9	1129	78.0	75.5 to 80.4
I would be prepared to use the FIT kit again in the future	656	431	32	13	2	1134	95.9	94.9 to 96.9
I understand what the test is being used for	626	486	8	10	2	1132	98.2	97.3 to 98.9
I think about the future of my health and this influences my behaviour today	616	436	62	10	1	1125	93.5	91.9 to 94.8
The ability of the test to detect cancer is important for me in making the decision to complete the test	795	303	25	4	2	1129	97.3	96.1 to 98.1
The ability of the test to detect pre-cancerous lumps is an important factor for me in making the decision to complete the test	782	308	28	3	3	1124	97.0	95.8 to 97.8

^a^Depending on which is the larger number. FIT = faecal immunochemical test.

## Results

### Patient responses

Packs with questionnaires were sent to 3760 patients taking part in the NICE FIT study; 1151 (30.6%) questionnaires were returned and analysed. Table 3 shows the questionnaire and FIT kit response rates from patients inside of London compared with those outside of London. A total of 1051 (91.3%) questionnaires were answered completely and the remaining 100 partially completed. Partially completed questionnaire responses were included in the study. Of the 1151 patients who returned questionnaires, 1142, (99.2%) also returned a FIT. There were nine patients who returned the questionnaire without returning the FIT kit. They were included in the analysis as they had the opportunity to physically examine the FIT kit, which was sent to them with the questionnaire. Of the 1142 patients who returned FIT for laboratory analysis with the questionnaire, 1126 (98.6%) produced a faecal sample that could be processed to give a f-Hb result.

**Table 3.** Questionnaire response rates inside of London compared with outside of London

Screening and responses	Overall	London sites	Sites outside of London
Screening (FIT packs sent), *n*	3760	2366	1394
Questionnaires returned, %	1151 (30.6)	408 (17.2)	743 (53.3)
FIT tests returned, %	1367 (36.4)	509 (21.5)	858 (61.5)

FIT = faecal immunochemical test.

### Patient characteristics

Demographics of responders are shown in Table 4. The mean age of responders was 65 years, 54.6% (*n* = 617) of patients were female, 88.0% (*n* = 985) from a white ethnic group, 94.9%% (*n* = 1072) preferred language was English, and 71.7% (*n* = 825) had previously used a ‘stool’ test.

**Table 4.** Demographic characteristics of patients who responded to questionnaire

Characteristic	Variable	*n* (%)^a^	Missing, *n* (%)
Sex	Male	514 (45.4)	20 (1.7)
	Female	617 (54.6)	
Age, years, and sex	25–39 (Male : Female)	13 : 15 (1.1 : 1.3)	10 (0.9)
	40–64 (Male : Female)	199 : 254 (17.4 : 22.3)	
	≥65 (Male : Female)	296 : 364 (25.9 : 31.9)	
Preferred language	English	1072 (94.9)	21 (1.8)
	Other	58 (5.1)	
Ethnic group	White	985 (88.0)	32 (2.8)
	Non-white	134 (12.0)	
Previous stool test experience?	Yes	825 (71.7)	1 (0.09)
	No	325 (28.3)	
Deprivation index	IMD 1–5 (more deprived)	509 (47.4)	78 (6.8)
	IMD 6–10 (less deprived)	564 (52.6)	

^a^Percentage calculated from the total number of responses for each category. IMD = Index of Multiple Deprivation.

### Characteristics of patients who did not respond to the questionnaire

Table 5 shows demographic information for responders and non-responders in London. Responders were slightly older than non-responders (mean age 64 years compared with 61 years), but no significant differences were found by sex or deprivation.

**Table 5.** Comparison of demographic characteristics of responders and non-responders (London)

Characteristic	Variable	Non-responders, *n* (%)	Responders, *n* (%)	% of records available for the non-responders	*P* value
Sex	Male	867 (46.0)	194 (47.9)	96.2	0.26^a^
	Female	1017 (54.0)	211 (52.1)		
Deprivation index	More deprived (IMD 1–5)	814 (43.2)	198 (43.9)	96.2	0.79^a^
	Less deprived (IMD 6–10)	1070 (56.8)	253 (56.1)		
Age, years	<39	82 (7.1)	14 (3.5)	59.0	0.003^b^
	40–64	572 (49.5)	187 (46.5)		
	>64	501 (43.4)	201 (50.0)		

^a^χ^2^ test, two-sided *P* value. ^b^ *t*-test. IMD = Index of Multiple Deprivation.

### Feasibility of FIT

Over 90% of patients felt FIT was practical, agreeing that it was straightforward to collect the faecal sample (90.2%; 95% CI = 88.3% to 91.8%), the device was easy to open and close (95.9%; 95% CI = 94.6% to 96.9%), and the instructions were easy to understand (96.3%; 95% CI = 95.1% to 97.3%). In addition, 78.1% (95% CI = 75.6% to 80.4%) agreed that they would prefer FIT to colonoscopy, and 90.5% (95% CI = 88.6% to 92.0%) would prefer returning FIT through the post (Supplementary Figure S1, Table 2). Of the 9 patients in the study who did not return the FIT but responded to the questionnaire, 8 agreed it was straightforward to collect the faecal sample (88.9%).

### FIT and faecal aversion

It was found 76.3% (95% CI = 73.7% to 78.6%) of patients disagreed using FIT was unhygienic, 77.0% (95% CI = 74.9% to 79.4%) disagreed it was difficult to overcome disgust related to stools, and 79.2% (95% CI = 76.7% to 81.4%) disagreed it was difficult to overcome embarrassment using FIT (Supplementary Figure S2, Table 2). 8 out of 9 patients (88.9%) who did not return FIT but responded to the questionnaire disagreed FIT was uhygienic.

### Patient self-assessment of bowel cancer knowledge

Regarding bowel cancer knowledge, 78.0% (95% CI = 75.5% to 80.4%) of patients were worried about getting CRC (referred to as ’bowel cancer’ in questionnaire), 93.0% (95% CI = 91.4% to 94.4%) felt that there was a good chance of cure if detected early, and 75.1% (95% CI = 72.5% to 77.5%) felt that having a family history of CRC increased their risk (Supplementary Figure S3, Table 2).

### Statements relating to future test intentions

On future test intentions, 97.3% (95% CI = 96.1% to 98.1%) of patients felt that the ability to detect cancer was important for them when deciding to use FIT. It was found 95.9% (95% CI = 94.9% to 96.9%) would use the test again and 98.2% (95% CI = 97.3% to 98.9%) understood what the test was being used for. In addition, 93.5% (95% CI = 91.9% to 94.8%) agreed with the statement 'I think about the future of my health and this influences my behaviour today' (Supplementary Figure S4, Table 2). 8 out of 9 patients (88.9%) who did not return FIT but responded to the questionnaire agreed they would use FIT again.

### Analysis of responses in relation to covariates

Supplementary Table S1 shows ORs for key patient questionnaire responses using logistic regression and Table 6 shows patient numbers within variable groups. Significant differences in FIT perception were found. Patients who responded to the questionnaire and who returned a FIT that was successfully analysed to produce an f-Hb result, were four times more likely to find it straightforward to collect their stool sample (OR 4.29; 95% CI = 1.31 to 14.08) and four times more likely to prefer to use FIT rather than undergo a colonoscopy (OR 4.32; 95% CI = 1.49 to 12.52). Patients between 40 years and 64 years were less likely to find it straightforward to collect a stool sample than patients aged >65 years (OR 0.58; 95% CI = 0.36 to 0.93) and less likely to prefer FIT over colonoscopy (OR 0.60; 95% CI = 0.43 to 0.84). Patients in London were half as likely as those outside of London to prefer to use FIT than undergo colonoscopy (OR 0.50; 95% CI = 0.36 to 0.71). Willingness to use FIT in the future was stronger in patients who successfully used FIT (OR 11.08; 95% CI = 2.74 to 44.75), those from white compared with non-white backgrounds (OR 3.20; 95% CI = 1.32 to 7.75), and those with previous faecal test experience (OR 2.06; 95% CI = 1.03 to 4.13). No differences were seen in patients’ perception of FIT hygiene across groups. Patients from more deprived backgrounds were less likely to say that early detection of bowel cancer could be curative (OR 0.58; 95% CI = 0.35 to 0.98).

**Table 6.** Numbers of patients within variable groups used in logistic regression^a^

Characteristic	Variable	Frequency, *n*	%
Test used properly	Yes	947	98.4
	No	15	1.6
Deprivation	IMD 1–5	457	47.5
	IMD 6–10	505	52.5
Area	London	386	40.1
	Outside of London	576	59.9
Age, years	25–39	25	2.6
	40–64	388	40.3
	≥65	549	57.1
Sex	Male	435	45.2
	Female	527	55.8
Preferred language	English	908	96.6
	Non-English	54	5.4
Ethnic group	White	838	87.1
	Non-white	124	12.9
Previous stool test experience	Yes	681	70.8
	No	281	29.2

^a^In total, 962 responses were used in the logistic regression as not every questionnaire item was answered by every patient. IMD = Index of Multiple Deprivation.

## Discussion

### Summary

To the authors’ knowledge, this is the first study to determine patient perception of FIT in patients with suspected CRC symptoms. In this study, FIT was acceptable to most symptomatic patients who responded; most patients who used FIT felt it was easy to find time to use it and it was hygienic. Despite this, differences in FIT perception were seen between groups; for example, age group differences were seen, with patients aged between 40 and 64 years less likely to find the test straightforward and less likely to prefer FIT over colonoscopy compared with those aged >65 years. The study found some variation by geography and ethnic groups; for example, willingness to use FIT again was stronger in patients from white compared with other non-white groups, and in those outside London. This did not appear to be based on differences in hygiene perception. Patients who used FIT correctly reported finding the test more straightforward than those who did not and were more likely to prefer FIT over colonoscopy.

### Strengths and limitations

The study provides an insight into attitudes and perception of over 1000 symptomatic patients who had the opportunity to use FIT, a group whose views should be considered when designing and introducing new FIT pathways. To the authors’ knowledge, the only other study to address this issue was by von Wagner *et al* who asked public volunteers to imagine they had CRC symptoms, and found 70% would prefer FIT to colonoscopy if the risk of missing cancer was 1%.^15^ In the present study of truly symptomatic patients, 78.1% preferred FIT to colonoscopy.

The response rate to the survey was 30.6%, and consent to participation was through questionnaire return; those patients who did not return it were considered as not wishing to participate and were not contacted further. Characteristics of non-responders were only available for the London part of the sample owing to centralised recruitment in London; however, comparison of responders and non-responders showed only minor differences with responders being slightly older. There are many possible reasons for patients choosing not to participate, chief among them could be the voluntary nature of research studies. It would have been useful to include the views of those who did not respond, and this is the subject of a separate study being planned. Similarly, using NICE FIT infrastructure to rapidly disseminate questionnaire packs meant that questionnaires were only able to be sent in English and translation into more languages, which would have allowed a broader range of responses, could not be accommodated. Good acceptability in this study could be explained by different attitudes of the symptomatic population, who would be motivated to use tests to help diagnose their potential CRC symptoms, but also could be explained by those who participated being more likely to give a FIT sample. However, in the nine patients in the study who did not return the FIT sample but completed the questionnaire, eight out of nine patients agreed FIT was straightforward to use, was not unhygienic, and agreed they would use FIT again, consistent with overall study findings. Only a small number of patients, 15 (1.6%), were not able to use the FIT kit successfully and completed the questionnaire; therefore, ORs relating to this variable need to be interpreted with caution. In addition, only 5.4% of patients preferred a non-English language and 12.9% were from a non-white ethnic group.

Questionnaires were sent to patients as they were recruited to the NICE FIT study, therefore questions relating to investigation outcomes were not asked as some patients would not have diagnostic outcomes if they responded promptly. Further studies exploring how colorectal investigation outcomes affect FIT perception would be valuable. In addition, although all patients had urgent 2WW symptoms, the questionnaire did not categorise these symptoms further to determine if specific bowel symptoms affected FIT perception.

### Comparison with existing literature

Negative feelings of faecal interaction are a recognised barrier to faecal tests offered as part of screening programmes in asymptomatic people;^7^ this is not necessarily transferable to testing in a diagnostic pathway. However, poor uptake has also been reported in symptomatic patients with chronic conditions, such as inflammatory bowel disease, who were asked to provide a stool sample for faecal calprotectin measurements to monitor disease.^16^ Hygiene concerns and embarrassment have previously been reported in patients asked to provide faecal samples by their GP.^17^ For this reason, it is important to better understand variation in attitudes to using FIT, patient experience of using FIT, and interaction with the faecal sample. The study has found that faecal aversion was not associated with FIT use by most symptomatic patients who responded.

Differences in perception of faecal tests between patient groups have previously been considered by Orbell *et al*, who suggest that some ethnic minorities may have lower perceived health vulnerability beliefs that may affect test uptake.^18^ While differences were not found in hygiene perception between patient groups, or by deprivation index decile, as might be expected from previous studies,^19^ intention to use FIT in the future was greater in those from white compared to other non-white groups. There is no clear explanation for preference for FIT over colonoscopy in older patients and further studies to explore this would be valuable. The study found that previous experience of any faecal test, as would be more likely in older patients, doubled patient intention to use FIT, underlining that faecal aversion was not a barrier to intention to use FIT again.

### Implications for research and practice

This study highlights that there is variation in FIT perception between patients offered the test when experiencing suspected CRC symptoms. Incorrect FIT use affected preference for FIT over colonoscopy as an initial test, and addressing barriers to incorrect use, either owing to understanding or physical dexterity, is important at primary care and secondary care levels both to reduce the number of times the test is repeated to avoid diagnosis delay and to retain patient willingness to use FIT. Patient preference for postal test return rather than via primary care should be considered when designing FIT pathways to help deliver patient-centred care. At the same time, in developing these pathways one can be confident that most patients find FIT highly practical and acceptable. Further studies to determine patient decision-making behaviour, particularly in terms of CRC risk perception in relation to FIT result, in the context of FIT pathways, would be valuable. Qualitative studies assessing patient perception of FIT through interviews are also needed to gain greater depth of understanding of differences in patient responses to FIT.

In conclusion, patients presenting with suspected CRC symptoms who used FIT in this study found FIT practical, hygienic, and most would use it again. However, perception differences were seen in patient groups from ethnic minority backgrounds and older compared to younger patients.

## Clinical Trial Registration

ISRCTN49676259
